# Microvascular Invasion in Hepatocellular Carcinoma: A Review of Its Definition, Clinical Significance, and Comprehensive Management

**DOI:** 10.1155/2022/9567041

**Published:** 2022-03-30

**Authors:** Zehao Zheng, Renguo Guan, Wang Jianxi, Zhen Zhao, Tianyi Peng, Chunsheng Liu, Ye Lin, Zhixiang Jian

**Affiliations:** ^1^Shantou University Medical College, Shantou, China; ^2^Department of General Surgery, Guangdong Provincial People's Hospital, Guangdong Academy of Medical Sciences, Guangzhou, China; ^3^Sun Yat-Sen University Cancer Center, State Key Laboratory of Oncology in South China; Collaborative Innovation Center for Cancer Medicine, Guangzhou 510060, China; ^4^The Second School of Clinical Medicine, Southern Medical University, Guangzhou, China; ^5^Department of General Surgery, School of Medicine, Southern China University of Technology, Guangzhou, China

## Abstract

Hepatocellular carcinoma (HCC) is one of the most common types of malignancies in the world, and most HCC patients undergoing liver resection relapse within five years. Microvascular invasion (MVI) is an independent factor for both the disease-free survival and overall survival of HCC patients. At present, the definition of MVI is still controversial, and a global consensus on how to evaluate MVI precisely is needed. Moreover, this review summarizes the current knowledge and clinical significance of MVI for HCC patients. In terms of management, antiviral therapy, wide surgical margins, and postoperative transcatheter arterial chemoembolization (TACE) could effectively reduce the incidence of MVI or improve the disease-free survival and overall survival of HCC patients with MVI. However, other perioperative management practices, such as anatomical resection, radiotherapy, targeted therapy and immune therapy, should be clarified in future investigations.

## 1. Introduction

Hepatocellular carcinoma (HCC) is the most common malignant cancer in the world and accounts for 80% of primary liver tumours [1]. In China, HCC is the second leading cause of cancer-related death and ranks third in terms of incident tumour cases after lung cancer and gastric cancer [2, 3]. From 1990 to 2017, the mortality of HCC increased by 50% (from 20 to 30 deaths per 100,0000) [4]. Currently, various therapies have been applied to treat HCC. Several guidelines have recommended the following main treatments: surgical therapies (including surgical resection and liver transplantation), tumour ablation, transcatheter arterial chemoembolization (TACE), targeted therapy, and immunotherapy [5–8]. Most of these treatments can achieve some prolongation of survival but still remain unsatisfactory because of the high recurrence and metastasis rate [9–11]. Almost 70% HCC patients undergoing liver resection will relapse within 5 years [12]. Studies have shown that microvascular invasion (MVI) may be occult micrometastasis of the primary tumour, which can cause early recurrence in HCC patients undergoing surgical resection and lead to a poor prognosis [13–15]. The incidence of MVI differs from 15%-60%, which may be caused by the different diagnostic criteria of MVI and sampling methods for specimens in different studies [16].

The prevention of MVI and the treatment of HCC patients with MVI are still controversial. A multicentre study revealed that anatomical resection is a positive prognostic factor for HCC patients with MVI [17]. However, Lee et al. [18, 19] reported that anatomical resection could not improve the prognosis for patients with HCC >2 cm with MVI. Moreover, Yang et al. pointed out that preoperative TACE had no effect on the incidence of MVI [20]. But Chen et al. found that postoperative adjuvant TACE were beneficial for those HCC patients [21]. The subgroup analysis of a three-phase RCT showed that administering sorafenib treatment for those HCC patients did not improve their DFS [22]. However, several retrospective studies suggested that surgical resection plus sorafenib treatment could greatly improve the prognosis of the MVI patients [23, 24].

Therefore, this review starts with a description of the definition of MVI and comprehensively summarizes the clinical significance of MVI in HCC patients. In addition, based on the currently published research, this review provides an update on the current perioperative treatments available for HCC patients with MVI.

## 2. Methods

We searched MEDLINE/PubMed and Web of Science databases with Medical Subject Headings (MeSH) and non-MeSH terms (Supplementary Table 1) and without any restrictions from January 2008 to July 2021. The search resulted in 3377 articles related to these topics. Case reports, letters, articles related to MVI predictions, mechanism, reviews, and duplicated articles were excluded after Z-ZH and G-RG carefully read the titles and abstracts. Randomized controlled studies, prospective and retrospective studies, and meta-analysis were included in these reviews.

## 3. Definition of Microvascular Invasion

**Figure 1:**
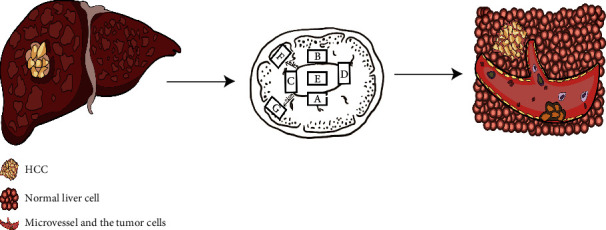
Chinese pathological diagnosis guidelines recommended that all liver cancer specimens should be sampled based on the 7-point baseline sample collection protocol. MVI is a nest of malignant cells in vessels lined with endothelial cells only visible under a microscope.

Before 2012, there were large differences in the incidence of MVI in HCC among several retrospective studies due to a lack of consensus on the definition of MVI. Professor Manuel and his teams performed a systematic review and proposed a definition for MVI. The systematic review pointed out that MVI is a nest of malignant cells in vessels (arteries, hepatic vein, and portal vein) lined with endothelial cells only visible under a microscope. In particular, they highlighted that based on existing research, the invasion of small arteries or lymphatic vessels should also be considered MVI [16]. In 2015, Chinese pathologists and clinical experts reported the first practice guidelines for the pathological diagnosis of primary liver cancer. They recommended that all liver cancer specimens should be sampled based on the 7-point baseline sample collection protocol to make a more accurate pathological diagnosis of primary liver cancer. Their guidelines also noted that MVI should be evaluated in all tissue sections, and the MVI grading system was stratified into three levels: **(**1**)** M0: no MVI; (2) M1 or low risk: MVI <5 vessels and ≤1 cm away from the adjacent peritumoural liver tissue; and (3) M2 or high risk: MVI >5 vessels or >1 cm away from the adjacent peritumoural liver tissue (Figure 1). The difference was that Chinese pathologists believed that immunohistochemical staining should be applied to confirm the vascular nature, and small arteries, bile ducts, and lymphatic vessels should be reported [25, 26]. Moreover, the Liver Cancer Pathology Group of China (LCPGC) conducted a nationwide multicentre study (patients = 16144) and verified the efficacy and accuracy of the seven-point sampling protocol (SPSP) and three-tiered MVI grading (MVI-TTG) scheme [27].

According to “the General Rules for the Study of Primary Liver [2017 edition]” [28], microvascular invasion (MVI) in Kang's hospital has been recorded as microvessel invasion (MI), microscopic portal vein invasion (MPVI), microscopic hepatic vein invasion (MHVI), and hepatic artery invasion (MHAI). Microvessel invasion was defined as newly developed microvascular structures in the tumour capsule or compressed and fibrotic peritumoural nonneoplastic liver tissue. The prognosis of the MPVI group was poorer than that of the MI-only group in their research. Therefore, they believed that microvascular invasion in liver cancer specimens should be divided into MI and MPVI [29]. Chen et al. considered that the number of sampling sites (NuSS) and sampling location would affect the detection rate of MVI and finally have a negative impact on the prognostic evaluation. Therefore, they concluded that the number of sampling sites (NuSS) and sampling location needs to be adjusted according to the tumour size [30].

Thus, the current definition and method of MVI detection are still controversial.

## 4. The Clinical Significance of MVI in HCC Patients with Liver Resection

The recommended treatments for early-stage HCC patients in several guidelines are surgical resection, liver transplantation, and local ablation therapies. However, considering medical expenses and safety, surgical resection is still the preferred treatment option. Based on a retrospective study, including 425 HCC patients undergoing surgical resection, Lauwers et al. reported that 51% of patients was pathologically diagnosed with MVI, and MVI was identified as an independent prognostic indicator of HCC patients after surgery [31]. Lim et al.'s study indicated that without MVI, HCC patients beyond the Milan criteria can achieve an overall survival (OS) similar to that of patients who meet the Milan criteria [32]. Rodríguez-Perálvarez et al. conducted a meta-analysis of 4 observational studies (*n* = 1501) and concluded that MVI was a risk factor for the 3-year (RR = 1.82, 95% CI: 1.61–2.07) and 5-year RFS (RR = 1.51, 95% CI =1.29–1.77) of HCC patients undergoing liver resection [16]. Based on 14 studies, including 3033 HCC patients, a meta-analysis from Chen et al. concluded that MVI was an independent risk factor for poorer RFS and OS for solitary small HCC (diameter <5 cm) after hepatectomy [33]. In these 14 studies, the prevalence of MVI in HCC ranged from 12.4% to 46.5%. The OS and DFS of the MVI-positive group were significantly poorer than those of the MVI-negative group (OS: HR = 2.39, 95%CI = 2.02–2.84; DFS: HR = 1.79, 95%CI = 1.59–2.02). Although many studies have reported that MVI plays an important prognostic role in HCC, Huang et al. proposed different views. A study conducted by Huang et al. included 1540 patients who underwent radical resection and divided them into several groups according to the BCLC staging system. After multivariate regression analysis, the results suggested that MVI was an independent risk factor for OS (HR = 1.431, 95% CI, 1.163–1.761, *P* < 0.01) and RFS (HR = 1.400, 95% CI, 1.150–1.705, *P* < 0.01) in HCC patients with BCLC stage A disease and an independent risk factor for RFS in HCC patients with stage B disease (*P* = 0.043). Therefore, they believed that MVI had little prognostic value for BCLC stage 0 and B patients. [34] However, we found that in this study, there were only 163 HCC patients with BCLC stage B disease. The conclusion of Han et al.'s research was contrary to the conclusion of Huang et al.'s research. In Han et al.'s article, the result after PSM was similar to that without PSM. The RFS and OS of the MVI-positive group were significantly poorer than the RFS and OS of the MVI-negative group. Therefore, they recommended that hepatectomy was reasonable for HCC patients with BCLC B stage without MVI [35]. Recently, a retrospective study of multinodular HCC based on the SEER database (*n* = 5249) and a large Japanese cohort (*n* = 1175) suggested that regardless of the tumour size and the tumour number, MVI was an important independent prognostic factor for HCC patients after hepatectomy [36].

Recent studies have indicated that MVI still has important clinical significance for recurrent HCC. Peng et al. reported that MVI at primary tumours could be a predictor of response for the combined treatment of sorafenib and transarterial chemoembolization (TACE) for patients with recurrent intermediate-stage hepatocellular carcinoma. In their research, recurrent patients with MVI would benefit from combined regimens [37]. According to Chen et al. and Sun et al., MVI status in primary hepatectomy could help recurrent HCC patients in selecting the best locoregional treatments [38, 39]. Moreover, diagnosed MVI after repeated hepatectomy still had a negative impact on the RFS and OS of recurrent HCC patients [40, 41].

At present, we believe that MVI is a crucial pathological factor and has important clinical significance for primary and recurrent HCC patients. HCC patients undergoing hepatectomy should receive a completely pathological assessment and should be closely monitored to determine recurrence early and prolong long-term survival when they are diagnosed with MVI.

## 5. The Comprehensive Management of Microvascular Invasion

### 5.1. Neoadjuvant Treatment to Decrease Microvascular Invasion

According to the existing studies, antiviral therapy, preoperative TACE are the major neoadjuvant therapies for HCC patients to prevent the MVI. The characteristics of related articles are listed in Table 1.

#### 5.1.1. Antiviral Therapy

Previous studies have shown that patients with HBV-related HCC have a higher incidence of MVI [42–44]. A high preoperative HBV virus DNA load is an independent risk factor for MVI [45, 46]. In the study of Li et al., the incidence of MVI was compared between HCC patients who received at least one type of antiviral drug for more than 90 days before surgery and those who received surgery directly. Li et al. reported that compared with the nonantiviral treatment group, the neoadjuvant antiviral treatment group was associated with a lower incidence of MVI [46]. Wang et al. systematically reviewed the published literature on preoperative antiviral treatment of HBV-related HCC and tried to evaluate its association with the incidence of MVI. All of these studies included HBV-related HCC patients with or without operative antiviral therapy and detected MVI after hepatectomy. The results indicated that compared with nonantiviral treatment, preoperative antiviral treatment could reduce the relative risk of MVI in HBV-related HCC patients by 40% [47]. However, the mechanism of how antiviral therapy can reduce microvascular invasion is unclear. Studies have shown that pERK was activated in HCC patients without antiviral therapy but not in antiviral patients, indicating that antiviral treatment could reduce MVI occurrence by affecting the activation of the MAPK/ERK signalling pathway.

Therefore, antiviral therapy may help prevent MVI formation and improve the poor prognosis of HBV-related HCC. Although these 6 studies were all non-RCTs, combined with previous high-quality studies showing that antiviral therapy could help reduce the incidence of primary liver cancer [48, 49], we are confident that antiviral therapy is important to prevent MVI formation.

#### 5.1.2. Preoperative TACE

TACE is currently an effective treatment for intermediate-stage hepatocellular carcinoma recommended by several guidelines [5–8]. The research conclusions of Li et al. suggested that receiving at least one preoperative TACE treatment can improve OS and RFS after hepatectomy for huge HCC (diameter ≥10 cm) [50]. Retrospective studies conducted by Zhou et al. [51] and Guo et al. [52] showed that for HCC patients with BCLC stage B disease, preoperative TACE combined with liver resection might achieve better oncology outcomes than TACE or liver resection alone but did not significantly increase perioperative complications. However, other studies have failed to show any significant survival benefit for preoperative TACE. A meta-analysis of 21 articles suggested that preoperative TACE did not seem to improve the prognosis of early-stage HCC patients [53]. Therefore, Yang et al. investigated whether preoperative TACE could reduce the incidence of MVI. They further screened the enrolled patients through propensity score matching and then performed multivariate regression analysis. The results showed that preoperative TACE had no effect on the incidence of MVI. Meanwhile, their results also showed that preoperative TACE had little influence on the DFS and OS of HCC patients after liver resection [20].

At present, there is no evidence supporting that preoperative TACE can reduce the incidence of MVI. Most studies have shown that preoperative TACE treatment does not improve the prognosis of early-stage HCC patients. Therefore, we do not recommend preoperative TACE treatment to reduce MVI formation.

### 5.2. Surgical Management for HCC Patients with a High Risk of MVI

#### 5.2.1. RFA Cannot Cure HCC Patients with a High Risk of MVI

Radiofrequency ablation (RFA) has been regarded as another first-line treatment option for small hepatocellular carcinoma by many guidelines [5–8]. The results of many studies have proven that RFA for small hepatocellular carcinoma can achieve the same overall survival rate as surgical resection, although it may be prone to cause residual cancer and have a poorer RFS [54]. Can RFA cure HCC patients with MVI? Considering that RFA treatment could not fully evaluate the specimens, Imai et al. screened three independent predictors of MVI and established an MVI prediction model [55]. The results showed that among patients receiving RFA, patients with 2–3 independent risk factors for MVI had a significantly poorer OS, and the RFS was shorter than that of patients with 0–1 independent risk factor. A study from South Korea reached similar conclusions [56]. By establishing an MVI prediction model, Li et al. first divided the patients receiving RFA treatment into high-risk and low-risk groups and then conducted propensity analysis and KM analysis. The results suggested that patients in the RFA treatment group had significantly shorter DFS than patients in the anatomical liver resection group (2-year RFS rate: 30.6% vs. 90.0%; HR: 4.83; 95% CI: 2.21–10.54). Finally, they concluded that patients with small hepatocellular carcinoma with MVI are not suitable for RFA. However, these two studies also had shortcomings. First, they were both retrospective studies with small sample sizes. Using only a small sample of data to establish the MVI prediction model can affect the reliability of the grouping. Second, the follow-up time of these studies was relatively short and provided only information on tumour-free survival. These factors all affected the generalizability of the conclusion. A large-sample multicentre prospective study is still needed.

#### 5.2.2. Anatomic Liver Resection for HCC Patients with a High Risk of MVI

For most early-stage HCC patients, surgical resection is still the best therapeutic method. Liver resection can be classified into nonanatomical resection (NAR) and anatomical resection (AR) [57]. Anatomic resection can completely remove the liver segment where the tumour is located, thereby reducing the probability of tumour recurrence and achieving better oncologic outcomes [58, 59]. A multi-institutional retrospective study of 546 HCC patients with microportal invasion from Japan indicated that HCC patients with microportal invasion receiving AR did not have a better RFS or OS than those receiving NAR [60]. Similarly, Jung et al. [18, 19] reported that in patients with HCC >2 cm and MVI, AR could not remove transportal-spreading tumour cells or improve prognosis. Recently, a multicentre retrospective study on HCC patients with MVI from China (including 1517 patients in 5 hospitals) was also published [17]. The results revealed that only one-third of these patients with MVI achieved a long-term survival of 5 years. Multivariate analysis suggested that anatomical resection is a positive prognostic factor (OR: 0.701, 95% CI: 0.535–0.919). Sun et al. conducted a meta-analysis of 12 retrospective studies to compare the safety and oncology outcomes of anatomical resection and nonanatomical resection for HCC patients with MVI [61]. Based on 7 studies, the 5-year survival time of AR was longer than that of NAR (RR: 0.76, 95% CI: 0.65–0.89, *P* < 0.01). Based on 12 studies, AR conferred a significant advantage for the overall HR of DFS (HR: 0.64, 95% CI: 0.45–0.91, *P* < 0.05). Unfortunately, these two studies did not perform subgroup analyses according to tumour size. Recently, Hu et al. performed an interesting study [62]. First, they established and verified the prediction model of MVI for HCC patients. Then, the HCC patients were divided into a high-risk group and a low-risk group using the MVI prediction model, and then, the impact of anatomical resection and nonanatomical resection on the prognosis was compared. The results suggested that regardless of the tumour size, for HCC patients in the high-risk group of MVI, the recurrence and mortality rates in the anatomical resection group were lower than those in the nonanatomical resection group.

Taken together, these conclusions seem to conflict with each other. The latest meta-analysis does not eliminate doubts about whether surgeons should perform surgical management based on tumour size for HCC patients with MVI. From the experiences of our team, anatomic liver resection is preferred for HCC patients with MVI who have sufficient residual liver volume and nonanatomical resection with adequate surgical margins could still be an alternative choice.

#### 5.2.3. The Importance of Surgical Margin Width in Nonanatomical Resection

If anatomical resection is not possible, how can we improve the prognosis of patients with MVI during surgery? In 2000, Poon et al. compared two groups of people with narrow (<1 cm) or wide (≥1 cm) surgical margins. They found that the width of the surgical margin did not affect the recurrence of HCC patients after surgery [63]. Subsequently, Shi et al. conducted a prospective study on 169 patients undergoing surgical resection. The results showed that patients with a wide margin (2 cm) had a significantly lower postoperative recurrence rate than patients with a narrow margin (1 cm) and could improve 5-year overall survival [64]. After conducting a meta-analysis of 7 studies on surgical margins, Zhong et al.concluded that compared with narrow margins (<1 cm), wide margins (≥1 cm) could significantly improve the prognosis of HCC patients (5-year OS: OR: 1.76, 95% CI: 1.20–2.59, *P* < 0.01; 5-year DFS: OR: 1.69, 95% CI: 1.37–2.08, *P* < 0.01) [65]. An international and multi-institutional study (including 404 T1 stage HCC patients) showed that resection margins >1 cm did not improve the oncologic outcomes of anatomical liver resection in patients with stage T1 disease. However, it could reduce the recurrence rate of T1 HCC patients undergoing nonanatomical resection [66]. Wang et al. divided 904 isolated HCC patients (diameter ≤5 cm) into a narrow margin group (<2 mm) and a wide-margin group (>2 mm) [67]. The results showed that a wide resection margin significantly improved the prognosis of patients with MVI. A retrospective study of 929 patients with MVI showed that the wide surgical margin (≥1 cm, *n* = 384) of patients with MVI had a better 5-year recurrence-free rate (71.1% vs. 85.9%, *P* < 0.001) and overall survival rate (44.9% vs. 25.0%, *P* < .001) [68] Han et al. published a multi-institution study on the impact of surgical margin and MVI on HCC patients after radical resection. According to the article, after performing multivariate analysis, narrow RM and positive MVI had the highest hazard ratio with reduced OS and RFS (OS: HR: 2.96, 95% CI: 2.11–4.17; RFS: HR: 3.15, 95% CI: 2.09–4.67) [69]. Therefore, some scholars suggested that for patients predicted to have a high risk of MVI before surgery, hepatectomy should be performed with a wide margin.

In summary, related studies about surgical methods mentioned above are listed in Table 2 and we believe that surgical methods have a significant impact on the DFS and OS of HCC patients with MVI. We should fully evaluate the sensitivity, specificity, and generalizability of the existing predictive model of MVI. Then, the preoperative evaluation model should be applied to select the high-risk group for MVI and choose the appropriate surgical methods.

### 5.3. Management of HCC Patients with MVI after R0 Liver Resection

Recently, postoperative adjuvant transarterial chemoembolization (pa-TACE), postoperative radiotherapy, and sorafenib are the major adjuvant therapies for patients with HCC with MVI. This part is divided into three sections and related articles are listed in Table 3.

#### 5.3.1. Transarterial Therapy

At present, transarterial therapies, mainly adjuvant transarterial chemoembolization and hepatic arterial infusion chemotherapy, are the main treatment options for patients with advanced unresectable hepatocellular carcinoma or those with high-risk prognostic factors after liver resection [5–8]. However, whether pa-TACE can reduce recurrence and improve the survival of HCC patients with MVI remains controversial. Sun et al. retrospectively analysed 322 HCC patients with MVI after liver resection [70]. Among them, 137 patients received TACE treatment in the 4th week after surgery. The results suggested that the early RFS, late RFS, and OS of MVI patients receiving pa-TACE treatment were better than those of MVI patients undergoing liver resection alone. Ye et al. retrospectively studied 519 HCC patients who underwent R0 surgical resection [71]. In their study, patients were divided into the MVI group (*n* = 260) and the without MVI group (*n* = 259). In the MVI group, compared with patients undergoing only liver resection (LR), patients who received LR+TACE treatment had significantly better RFS (median RFS: 37 months vs. 13 months) and OS (4 years, 67.5% vs. 53.9%). In the patients without MVI, there was no significant difference in RFS or OS between the LR+TACE group and the LR-only group. Interestingly, pa-TACE in this study was performed 1, 3, and 6 months after hepatectomy.

Recently, Chen et al. conducted a meta-analysis of 12 studies (including one RCT study and two prospective studies) on the prognostic value of pa-TACE for HCC patients with MVI [72]. The 5-year OS rates favoured postoperative TACE rather than HR alone (OR: 0.59; 95% CI: 0.48~0.73) in 9 of the included studies, with 838 patients undergoing HR plus postoperative TACE and 1078 patients undergoing only HR. Based on the results from 10 of the included studies, HCC patients with postoperative TACE had longer 3-year DFS (OR: 0.50; 95% CI: 0.41~0.60) and 5-year DFS (OR: 0.58; 95% CI: 0.46~0.73). However, it is worth noting that in these articles, the time, frequency, and drugs of TACE after surgery were not the same.

In summary, we believe that patients with hepatocellular carcinoma who undergo surgical resection should receive TACE treatment as soon as possible if MVI is considered for pathological diagnosis, especially for those patients with tumour diameters >5 cm and multiple nodules. TACE regimens depend on the recovery of the patient's systemic and hepatic functions.

#### 5.3.2. Radiation Therapy

Studies have shown that compared with TACE, postoperative radiation therapy may provide better RFS for HCC patients with MVI. One study retrospectively analysed 117 hepatitis B virus- (HBV-) related HCC patients with MVI [73]. Patients were divided into two groups based on postoperative adjuvant treatment (RT group and TACE group), and propensity score matching (PSM) was performed to adjust the differences in baseline characteristics. The results suggested that the RFS of the RT group was significantly longer than the RFS of the TACE group (25.74 ± 8.12 months vs. 9.18 ± 1.67 months). There was no significant difference in OS between the two groups.

One year later, the author published the results of a nonrandom study about RT therapy [74]. HCC patients with MVI were divided into an RT group (*n* = 29) and a control group (*n* = 30), and antiviral therapy was administered to the control group. The results indicated that compared with standard postoperative treatment, postoperative adjuvant radiotherapy after hepatectomy provided better RFS for HCC patients with MVI. However, no significant difference in OS was observed.

Recently, Yang et al. published a meta-analysis on postoperative adjuvant treatment for HCC patients with MVI [75]. They noted that compared with pa-TACE, postoperative radiotherapy could reduce the recurrence of HCC patients with MVI after radical resection. However, we found that this conclusion is based on the studies of Wang et al. Multicentre and large-sample prospective studies are needed to verify the conclusions. At present, our team believes that the current level of evidence for “RT is better than TACE for HCC patients with MVI” is clearly insufficient, and caution should be taken when interpreting the conclusions from these studies.

#### 5.3.3. Targeted Therapy

Current studies have shown that sorafenib and lenvatinib have excellent performance in improving the PFS and OS of patients with advanced unresectable hepatocellular carcinoma [76–78]. Several popular guidelines have regarded sorafenib and lenvatinib as first-line treatments for patients with advanced unresectable hepatocellular carcinoma. A three-phase RCT showed that administering sorafenib treatment for HCC patients after surgical resection did not improve their DFS but increased the incidence of side effects of sorafenib, such as diarrhoea, hand-foot skin reactions, and fatigue [22]. According to their definition, HCC patients with one tumour plus microvascular invasion, satellite tumours, poorly differentiated microscopic appearance, or multimodule tumours were defined as having a high risk of recurrence. An intermediate risk was defined as a single tumour (≥2 cm) with a well-differentiated or moderately differentiated microscopic appearance and MVI or satellite tumours. Subgroup analysis indicated that there were no significant treatment group differences with respect to the median time to recurrence or overall survival based on the risk of recurrence. A retrospective study containing a small sample of HCC patients with MVI after hepatectomy conducted by Huang et al. team revealed that after receiving sorafenib treatment, the 3-year DFS was 56.3%, and the 3-year OS was 81.3%, which was significantly better than those of the control group [23]. Another retrospective study of 728 HCC patients with MVI after surgical resection showed that after propensity analysis, the 5-year RFS of the sorafenib treatment group was 39%, which was significantly better than that of the only surgical resection group (19%). The 5-year OS in the sorafenib treatment group was 57%, which was significantly better than that of the surgical resection group (37%) [24].

After searching and screening the studies, Gu et al. conducted a meta-analysis of four studies on sorafenib treatment for MVI patients [79]. The results suggested that compared with surgical resection only, surgical resection plus sorafenib treatment could greatly improve the prognosis of HCC patients with MVI (HR 1.369, 95% CI 1.193~1.570). However, this meta-analysis had some shortcomings. First, all studies were retrospective studies rather than RCTs or prospective studies, which may easily cause selection bias. Second, the patients in these four studies were all Chinese, which also weakened the generalizability of the conclusions.

## 6. Conclusion and the Vision for the Future

First, considering that different sampling methods will have a great impact on the evaluation of MVI and the importance of MVI in evaluating and monitoring the prognosis of HCC patients undergoing surgical resection, it is important and necessary for pathologists to accurately diagnose MVI. Although Chinese pathologists already have clear and standardized guidelines to help with pathological sampling and evaluate MVI in HCC specimens, a global consensus regarding the diagnostic criteria of MVI is still needed, which could improve bias due to differences in these criteria. Second, many studies on predicting MVI in HCC patients have been published. However, we found that few high-quality models are actually applied in clinical practice. Prospective studies should be conducted to verify the clinical effectiveness and to determine high-quality models. These high-quality predictive models can indeed play a role in clinical practice and further improve the prognosis of HCC patients, such as guiding doctors and patients to choose better surgical methods. Third, for HCC patients pathologically diagnosed with MVI, postoperative TACE has been proven to be a useful choice in improving DFS and OS, so clinicians should recommend TACE treatment for MVI patients. Finally, in view of the good performance of targeted therapy and immunotherapy in improving the prognosis of advanced liver cancer, relevant prospective studies should be carried out in the future to clarify these prognostic improvements in the MVI subgroup when treated with these two therapies.

## Figures and Tables

**Table 1:** Neoadjuvant treatment to decrease microvascular invasion.

Author	Years	Study types	No. of study	No. of patients	Therapy	The rate of MVI (%)	OR	95% CI
Wang et al.	2020	Meta-analysis	6	4988	Antiviral therapy vs. surgery directly	/	**0.6**	**0.49-0.73**
Yang et al.	2021	Propensity score matching retrospective study	1	1624	Preoperative TACE vs. surgery directly	38.85% vs. 41.10%，*P* = 0.473	/	/

**Table 2:** Surgical management for HCC patients with a high risk of MVI.

Author	Years	Study types	No. of studies	No. of patients	Therapy	RFS (%)	OS (%)
Imai et al.	2018	Retrospective study	1	159	RFA (without MVI vs. with MVI)	5-year DFS 11.6% vs. 6.8%，*P* = 0.20	5-year 80.0% vs. 55.8%，*P* = 0.0037
Li et al.	2021	Retrospective study	1	516	RFA vs. SR	2-year 30.6% vs. 90.0%	/
Sun et al.	2021	Meta-analysis	12	1550	AR vs. NAR	5-year 37.72% vs. 27.51%, *P* < 0.001	5-year 61.7% vs. 59.17%, *P* < 0.001
Yang et al.	2019	Retrospective study	1	904	Narrow margin group (<2 mm) vs. a wide-margin group (>2 mm)	5-year 56.7% vs. 25.4%, *P* < 0.001	5-year 76.3% vs. 56.8%, *P* < 0.001
Han et al.	2020	Retrospective study	1	929	Narrow margin group (<1 cm) vs. a wide-margin group (>1 cmm)	5-year 71.1% vs. 85.9%, *P* < 0.001	5-year 44.9% vs. 25.0%, *P* < 0.001

RFA: radiofrequency ablation; MVI: microvascular invasion; SR: surgical resection; AR: anatomic resection; NAR: nonanatomical resection.

**Table 3:** Management of HCC patients with MVI after R0 liver resection.

Author	Years	Study types	No. of studies	No. of patients	Therapy	RFS (%)	OS (%)
Sun et al.	2015	Retrospective	1	322	Surgical resection plus TACE vs. surgical resection only	5 year DFS 35.0% vs.30.3%，*P* = 0.012	5-year 54.0% vs. 43.2%，*P* = 0.006
Ye et al.	2017	PSM and retrospective	1	260	Surgical resection plus TACE vs. surgical resection only	4 year DFS 30.9% vs 28.4%，*P* = 0.002	4-year 67.5% vs 53.9%；*P* = 0.019
Chen et al.	2020	Meta-analysis	12	2190	Surgical resection plus TACE vs. surgical resection only	5-year OR: 0.58; 95% CI: 0.46~0.73.	5-year OR: 0.59; 95% CI: 0.48~0.73
Gu et al.	2020	Meta-analysis	4	955	Surgical resection plus sorafenib vs. surgical resection only	HR 1.369, 95% CI 1.193~1.570	/

## Data Availability

The underlying data supporting the results of our study are provided in the article.

## Supplementary Materials

Supplementary Materials Supplemental Table 1. The data collection strategy of the review.
