# Supplementary material for: Microvascular Invasion in Hepatocellular Carcinoma: A Review of Its Definition, Clinical Significance, and Comprehensive Management
Source: J Oncol. 2022 Mar 30;2022:9567041. doi: 10.1155/2022/9567041 (PMC8986383; doi:10.1155/2022/9567041)
Supplement: Supplementary Materials — Supplemental Table 1. The data collection strategy of the review. [file 9567041.f1.docx]

**Supplemental Table 1. The data collection strategy of the review**

| PubMed/MEDLINE | Web of sciences |
| --- | --- |
| ("Carcinoma, Hepatocellular"[Mesh] OR hepatocellular carcinoma [Title/Abstract] OR liver cancer [Title/Abstract] OR Liver Cell Carcinoma [Title/Abstract] OR HCC [Title/Abstract]) AND (((microvascular [Title/Abstract] OR microvessel[Title/Abstract]) AND (invasion[Title/Abstract] OR emboli[Title/Abstract] OR thrombi[Title/Abstract] OR thrombosis[Title/Abstract])) OR (microemboli[Title/Abstract] OR microthrombi[Title/Abstract] OR microthrombosis[Title/Abstract]) ) | TS= (“hepatocellular carcinoma" OR "liver cancer" OR "HCC” OR "liver cell carcinoma" OR "hepatocellular neoplasm") AND TS= (microvascular OR microvessel) AND TS= ("invasion" OR "emboli" OR "thrombi" OR "thrombosis") |
